# Fatigue Crack Growth in Metallic Materials (Volume II)

**DOI:** 10.3390/ma19102063

**Published:** 2026-05-14

**Authors:** Fernando Ventura Antunes, Francisco Díaz Garrido

**Affiliations:** 1 Centre for Mechanical Engineering, Materials and Processes (CEMMPRE), ARISE, Department of Mechanical Engineering, University of Coimbra, 3030-788 Coimbra, Portugal; 2 Departamento de Ingeniería Mecánica y Minera, Campus de Las Lagunillas s/n, University of Jaén, 23071 Jaen, Spain; fdiaz@ujaen.es

## 1. Introduction

The in-service failure of critical components in cars, ships, aeroplanes, trains, etc., can lead to serious accidents, with major human and material consequences [1,2,3,4]. The lifetime of machines and structures should also be as long as possible for sustainability reasons, since the production of components needs energy consumption and emits industrial greenhouse gas emissions [5]. In service, the critical components are usually subjected to variable amplitude loading; therefore, their design against fatigue is fundamental to avoid failure. Moreover, there is an increasing pressure on designers to reduce the level of conservatism in engineering systems in order to meet targets on cost, weight, and emissions [6]. Weight is particularly important in components used in means of transport, such as planes or cars, since the highest environmental impact occurs during the use phase through fuel consumption.

The fatigue design strategy known as damage tolerance assumes the inherent presence of small defects resulting from the production process [7]. The inspection intervals of critical components are defined by calculating the propagation of the initial defects up to a critical length. This requires accurate values of the crack propagation rate. The present Special Issue is devoted to the study of fatigue crack growth (FCG) in metallic materials. In fact, critical components are most often produced using metallic materials, which justifies the emphasis on this class of materials.

FCG in metallic materials is a complex phenomenon, affected by several factors, namely, material parameters, loading, geometry, and environmental conditions (temperature and atmosphere) [8]. Depending on these parameters, different damage mechanisms are active at the crack tip. Cyclic plastic deformation is usually the main damage mechanism responsible for crack growth [9], but other mechanisms may come into action, like oxidation [10], cleavage [11], or the growth and coalescence of microvoids [12]. Crack closure, which is the contact of crack flanks during a portion of the load cycle, has a protective effect [13]. The study of FCG is carried out using experimental and numerical approaches, as is detailed next.

(i) Experimental analysis of FCG

Fatigue cracks are one of the main sources of structural failure in real in-service structures. The application of fracture mechanics to engineering design has enabled a more in-depth understanding of failure and crack growth mechanisms, but some aspects are still not fully elucidated. This lack of understanding arises principally from difficulties associated with quantifying the phenomenon and measuring its effect on the crack driving force.

Modern experimental techniques, such as Digital Image Correlation [14] or Differential Thermography [15], have actively contributed to a better understanding of the different fatigue and failure mechanisms. In particular, full-field techniques generally provide a very detailed description of the near crack tip stress field at the surface of the component [16,17].

From an experimental point of view, FCG has been commonly studied using the stress intensity factor range, ΔK, a linear elastic parameter, which relates the crack growth rate per cycle (da/dN) to ΔK in a log–log linear relationship [18]. However, da/dN-ΔK curves have limitations, such as sensitivity to stress ratio (R), inability to capture load history effects, and limited accuracy in predicting short crack behaviour. Moreover, ΔK is based on Linear Elastic Fracture Mechanics, the applicability limits of which are not always clearly defined, especially under conditions involving significant plasticity [19,20]. On the other hand, nonlinear parameters such as the cyclic plastic strain range [21], the dimensions of the reversed plastic zone [22], strain ratchetting [23], plastic CTOD [24], and plastic energy dissipation [25] have been the focus of many studies. These parameters directly quantify the plasticity at the crack tip and have shown consistent correlation with crack growth rates in a variety of loading scenarios, including constant and variable amplitude conditions, emerging as more reliable physical parameters governing crack advance. This has led to a growing emphasis on experimental methods with the capacity to capture localised plasticity fields, particularly under complex loading [26]. The characterisation of the near crack tip region is, however, particularly challenging due to its small size and high strain gradient.

One major advantage of full-field techniques is that they are generally non-invasive, allowing researchers to assess key parameters that are difficult to measure by conventional methods. However, a common feature of all investigations of fatigue crack growth involving full-field techniques is the need to accurately determine the crack tip position [27], since it is a critical variable that can significantly influence various aspects such as the experimentally inferred ΔK or COD. Experimental studies usually involve significant costs related to the preparation of specimens or components, testing and analysis of results. This is particularly relevant in full-scale tests, i.e., those involving machines like aeroplanes or structures. The vastness of parametric space makes it impossible to study it in experimental terms.

(ii) Numerical simulation of FCG

The numerical prediction of FCG allows for the development of systematic studies on loading, material, and geometrical parameters. After preparing the numerical model, changing the parameters is relatively easy. In addition, it permits a detailed view of stress and strain fields in the vicinity of crack front, where damage mechanisms act. The reduction in costs comparatively to the experimental approach and the accelerated development of products are among the key advantages of numerical simulation.

However, the ability to predict accurate values of da/dN is a complex task. There are four main complexities related to the modelling of material behaviour, the modelling of 3D geometries, the understanding of loading effects, i.e., the load history mechanisms, and the simulation of crack propagation.

The study of 3D geometries usually involves a huge number of finite elements, which easily turns into an unacceptable numerical effort [28,29]. A refined mesh is needed around the crack front to simulate the steep variations in stress and strain, which must encompass the crack tip plastic deformation region and the extension of crack propagation. This explains the dominance of 2D studies assuming plane stress or plane strain states.

Real load patterns typically have variable amplitudes and a huge number of loading cycles [30,31,32,33]. An unacceptable degree of numerical effort and time is involved in the direct simulation of these patterns. Therefore, most of the research focuses on constant amplitude loading or simple variable amplitude patterns like overloads, underloads, and load blocks. The simulation of real patterns is carried out using simplified strategies which implement empirical knowledge. Software like NASGRO [34,35], FASTRAN [36], AFGROW [37], or CRACK2000 uses ΔK-based crack propagation laws, namely, the Forman equation, along with retardation models to predict crack length [38,39]. However, their precision is unknown and questionable, and physically based models are recommended. Single loading usually results in mode I crack propagation, but multi-axial loading introduces additional complexities in terms of crack paths and crack growth rates.

The simulation of material behaviour is another huge challenge. Microstructural parameters play a major role, and these change with the material, even with different batches of the same material and heat treatment. The simulation of elastic–plastic behaviour is usually carried out using stress–strain cycles obtained in LCF tests [40]. However, the strain range is usually significantly lower than that observed at the crack tip, and therefore extrapolation is required with unknown accuracy. In addition, monotonic behaviour should be included, particularly in the presence of overloads. Additional mechanisms, acting synergistically or competitively, should also be included in the material model. At low load ranges, when the size of the cyclic plastic zone is smaller than the grain size, propagation usually is associated with cleavage, which is propagation along crystallographic planes. At relatively high load ranges, the initiation, growth, and coalescence of microvoids is relevant [41]. At relatively high temperature, diffusion-based mechanisms like oxidation and creep may become dominant, particularly under plane strain conditions, i.e., inside the components. Plasticity-induced crack closure is expected to be relevant at intermediate load levels, and its simulation is a natural consequence of cyclic plastic deformation and crack propagation. However, roughness-induced crack closure, more relevant at low load ranges, requires a complementary modelling strategy [42].

Finally, the parameters considered to define crack propagation must be linked to the damage mechanisms. If this is cyclic deformation, a parameter based on the accumulation of plastic deformation at the crack tip seems to be the most adequate. But this parameter needs to be modified to account for the effects of the environment or void growth.

Numerical simulation is used to predict crack propagation in real 3D components, submitted to real loading patterns, in a relatively short time. Ideally, damage prediction could be made after each mission, assuming the existence of a loading acquisition system. It should be noted that experimental results are always needed, at least for the validation of the numerical tools.

## 2. Contributions of This Special Issue

The present Special Issue makes a significant contribution towards a better understanding and modelling of FCG in metallic materials.

There has been a growing interest in additive manufacturing (AM) technologies in recent years as they present several potential advantages over traditional subtractive manufacturing. Mechanical properties are, however, influenced by porosity, internal defects, residual stresses, and microstructural heterogeneities resulting from the fabrication process. Sales et al. [43] studied FCG in additive manufacturing materials, produced using WAAM (Wire Arc Additive Manufacturing), where properties can be affected by porosity, residual stresses, and crack tip shielding mechanisms, which can be very different from those of conventionally manufactured materials. CT specimens with a thickness of 12.7 mm, made of duplex steel, were studied with crack propagation longitudinally and transversely to the WAAM deposition direction. A complex loading pattern was applied, consisting of five load blocks with constant maximum load, while the minimum load ranged between 0.095 kN (R = 0.01) and 4.75 kN (R = 0.5). Deposition direction was found to exert significant influence. When expressed in terms of the effective stress intensity factor range, ΔK_eff_, the fatigue properties in both directions were almost identical, which demonstrates the significance of crack closure mechanisms for AM materials. The authors believe that the difference in the crack tip opening values is due to the residual stresses induced by the rapid cooling nature of the WAAM bead layering process. The effect of crack closure on FCG is not consensual, but this study highlights its relevance. There is also some disagreement regarding the best method to determine the closure level.

The effect of porosity in AM materials, a main issue of this technology, was studied by Cerezo et al. [44]. The material studied was 18Ni300 maraging steel in the form of CT specimens, manufactured in SLM with different orientations, namely, 0°, 45°, and 90°. The circularity and aspect factors of the pores differed according to orientation, and a significant effect of orientation on FCG was also found.

Bogdanoff and Tiryakioglu [45] studied a hot isostatically pressed specimen of the A357 alloy in T6 condition, which was tested for fatigue performance in situ. CT specimens with W = 6.8 mm and a thickness of 1.8 mm were considered. Multiple small cracks became visible in the first cycle near and away from the stress concentration. Subsequently, these small cracks competed among them to become the main propagating crack. Some of these cracks coalesced and started propagating further, marking the end of the initial competition.

Gomes et al. [46] studied 51CrV4 chromium–vanadium alloyed steel, used in leaf springs, in the form of CT specimens with W = 35 mm and a thickness of 10 mm. The CT specimens were obtained from the LT and TL directions of suspension spring leaves, and Paris law and fatigue thresholds were obtained. Fracture surface analysis showed that the crack propagation process occurred predominantly in a transgranular way with micro-cleavage processes and without visible fatigue striations.

Martins et al. [47] studied compact tension (CT) specimens and two loading modes: pure mode I and mixed-mode loading (Modes I + II). FCG was simulated using the Extended Finite Element Method (XFEM), considering the Paris Law parameters of an AISI 316L stainless steel. The CT specimens had a thickness of 2.5 mm. Good agreement was found between the XFEM results for mode I loading and the a-N curve obtained experimentally. XFEM was also proven to be able to obtain the direction of crack propagation very effectively. These results demonstrate the suitability of the method developed to predict the fatigue life of more complex mechanical components in which there will be an initial crack, provided that the parameters of the material’s Paris Law are available. However, this approach assumes a linear elastic behaviour and therefore does not simulate crack closure.

Vasco-Olmo et al. [48] used the CJP model for the characterisation of the crack tip displacement fields. The CJP model has been developed to consider the influence of the plastic enclave generated around a fatigue crack by analysing its shielding effects on the surrounding elastic stress field. DIC was used to measure the experimental displacement fields in CT specimens made of 2024-T3 aluminium alloy and commercially pure titanium, with thicknesses of 1 and 2 mm, respectively. The CJP model defines three stress intensity factors for the characterisation of crack tip fields, namely, an opening mode stress intensity factor K_F_, a retardation stress intensity factor K_R_, and a shear stress intensity factor K_S_, and also provides the T-stress. The size and shape of the crack tip plastic zone were perfectly characterised by the CJP model through their comparison with experimental results obtained by DIC. The CJP model was thus demonstrated to be a valuable tool in fracture mechanics when plasticity plays a significant role, such as plasticity-induced crack shielding and crack tip plasticity. This study involved experimental work as well as analytical modelling of crack tip fields.

Neto et al. [49] studied the initiation and propagation of a crack crossing a hole, replicating the classical crack tip hole strategy to delay fatigue failure. The FCG rate was calculated using a node release strategy, assuming that cyclic plastic deformation is the main damage mechanism and that cumulative plastic strain is the crack driving parameter. The effects of plasticity-induced crack closure, crack tip blunting, material hardening, and residual stresses resulting from plastic deformation were included in the model in a natural way. Experimental work was developed in a CT specimen made of 7050-T7451 aluminium alloy, with a 3 mm diameter hole. Crack initiation life after the hole was estimated using the Theory of Critical Distances combined with the Smith–Watson–Topper parameter. A value of a0 = 31.83 µm was obtained for the El Haddad parameter, which was used to define the critical distance. Good agreement was found between the numerical predictions of da/dN and the experimental results, which validated the numerical approach and the dominance of cyclic plastic deformation. The main result, however, is the proposed methodology, which allows the prediction of initiation and propagation lives in notched components. The assumption that cyclic plastic deformation is the main mechanism responsible for FCG was also validated.

Nonlinear crack tip parameters were also used by Ajmal et al. [50], assuming once again that cyclic plastic deformation is the main damage mechanism responsible for FCG. They studied CT specimens made of 316L stainless steel. Their approach was based on correlating full-field displacement information with post-processing digital images. Their detailed post-processing protocol can be used to calculate plastic CTOD, and a linear relation was found between this parameter and da/dN.

Han et al. [51] studied Cu matrix composites, and in particular the effects of particle type, particle size, and particle distribution on fatigue behaviour. Soft particles with metallic bonding, which are cut by dislocations, and hard particles with ionic interatomic bonding, which are resistant to shearing by dislocations, were investigated. The materials studied were the commercially available Cu–alumina composite known as GLIDCOP^®^ and a commercially available Cr-particle-strengthened Cu alloy, CuCrZr_21017. Increasing the density of these particles enhanced the mechanical strength of the conductors, which the researchers tested under static loading. When the size of the strengthening particle was small, alumina had a greater strengthening effect than Cr. Conductors with higher mechanical strength exhibited lower electrical conductivity, and those strengthened by metallic particles exhibited less plastic deformation strain than those strengthened by ionic interatomic bonding particles. Atomic-scale shape corners induce higher stress concentration in conductors strengthened by ionic particles. In the presence of alumina particles, dislocations can be expected to curve around the particles.

Kujawski and Vasudevan et al. [52] studied the unusual lack of threshold in FCG behaviour observed at low values of ΔK for some alloys, loading conditions, and specimen geometries. This phenomenon, called Marci effect, was associated with the loading regime where only one threshold is satisfied, namely, when the applied K_max_ is larger than K_max,th_, whereas the applied ΔK is smaller than ΔK_th_. Moreover, the Marci effect of FCG in air is several times faster than that under vacuum, which indicates that some environmental factors assist the cracking process in lab air. Ni superalloys Ni100, Inconel 718, and Rene’95, which in general exhibit strong planar slip deformation, did not show the Marci effect. The authors hypothesised that the Marci effect is linked with time-dependent void generation (due to cross slip) under plane strain conditions for wavy slip alloys. This study shows the importance of microstructures and the environment, as well as reinforcing the idea that there are FCG issues that are still not understood.

In summary, various experimental and numerical studies were carried out and reported in the present Special Issue. A range of different materials were studied, namely, duplex steel produced by WAAM, 18Ni300 maraging steel produced by SLM, 7050-T7451 aluminium alloy, 2024-T3 aluminium alloy, AISI 316L stainless steel, 51CrV4 chromium–vanadium alloyed steel, and Cu matrix composites. Most of the studies involved mode I loading, but mixed-mode loading (I + II) was also investigated. Several experimental techniques were used, including DIC to quantify the displacement field around cracks, SEM to analyse the fracture surfaces, and energy-dispersive X-ray spectroscopy for the examination of elemental dispersion. Different crack driving parameters were also considered, namely, linear elastic parameters (ΔK, ΔK_eff_, Kmax, and the CJP parameters) as well as nonlinear parameters (plastic CTOD and cumulative plastic strain).

It is interesting how knowledge is built in small steps, without the need for absolute truths.

## 3. Future Research

Industries push the boundaries of performance, durability, and safety. Critical areas are transportation and energy. Additive manufacturing is emerging as a technological process enabling great flexibility in terms of geometry and even material. However, this production strategy introduces new complexities due to the presence of residual stresses and defects, and the variability of microstructures and properties even with small changes in processing conditions. The scatter associated with process parameters and heat treatments complicates the generation of reliable FCG databases. In this context, it is important to develop technical standards and guidance for AM structural integrity in order to generate databases of FCG results.

In terms of fundamental research, it is important to identify and model the fundamental damage mechanisms responsible for FCG in different materials, geometries, loadings, and environmental conditions. Cyclic plastic deformation is considered to be the main damage mechanism responsible for FCG, as was argued in various papers of the present collection. However, the effect of voids and the environment on this mechanism must also be understood.

In the numerical simulation of FCG, the final target is the ability to predict crack growth in real components submitted to realistic load patterns in a relatively short time, as already mentioned. The simulation of 3D cracked bodies usually involves a huge number of finite elements, and therefore it is fundamental to develop strategies for the remeshing of 3D geometries, in order to reduce the numerical effort to an acceptable level. The modelling of elastic–plastic behaviour must be improved with the inclusion of monotonic tensile results, which is important in variable amplitude loading. Moreover, the models must consider the possibility of competitive/collaborative damage mechanisms, namely, brittle failure (cleavage), roughness-induced crack closure, and the initiation, growth, and coalescence of microvoids. It is also important to understand the fundamental links between different areas of fatigue which are usually studied independently, namely, FCG, crack initiation, HCF (S-N curves), and LCF.

From an experimental point of view, an increase in the spatial resolution of the measuring systems will make it possible to increase the accuracy of measurements, contributing to a better understanding of FCG mechanisms. Moreover, the introduction of tomography in the study of fatigue crack growth may also generate new findings in the field.

The study of FCG has been based on analytical models fitted to a database of experimental results. However, the phenomenon of FCG has a large number of parameters and involves different physical processes, which means that these analytical models cannot easily combine all this complexity into a single mathematical expression. AI has now emerged as a tool able to accommodate all this complexity. A key difficulty, however, lies in the lack of high-quality, broadly applicable, material-level databases. Moreover, the parametric space is vast and there are significant regions without results; i.e., the databases are sparse, but models trained on sparse, condition-specific datasets tend to generalise poorly. The challenge, therefore, is the inclusion of the existing knowledge of physical damage mechanisms. Consequently, the future work proposed here requires significant collaboration between researchers devoted to fundamental studies, software developers, and component designers.
